# When a Bulbar Ulcer Hides a Darker Reality: The Unusual Diagnosis of a Portal Cavernoma in a 69‐Year‐Old Adult

**DOI:** 10.1002/ccr3.73040

**Published:** 2026-06-29

**Authors:** Martin Wendlassida Nacanabo, André Arthur Taryètba Seghda, Aimé Arsène Yaméogo, Ella Hatoula Lengani, Adolphe Benjamin Zingué Ouattara, Aminata Guiarra, Yannick Laurent Tchenadoyo Bayala, Abdoul Nassir Porgo, Abdoul Aziz, Abdoul Djalilou Traoré, Anna Tall/Thiam, Georges Christian Millogo, Valentin Nobila Yaméogo, André Koudnoaga Samadoulougou

**Affiliations:** ^1^ Cardiology Department Bogodogo University Hospital Ouagadougou Burkina Faso; ^2^ Cardiology Department Souro Sanou University Hospital Bobo Dioulasso Burkina Faso; ^3^ Cardiology Department Pala University Hospital Bobo Dioulasso Burkina Faso; ^4^ Rheumatology Department Bogodogo University Hospital Ouagadougou Burkina Faso; ^5^ Neurology Department Bogodogo University Hospital Ouagadougou Burkina Faso; ^6^ Rheumatology Department General Hospital Garoua Cameroon; ^7^ Internal Medicine Department Souro Sanou University Hospital Ouagadougou Burkina Faso; ^8^ Cardiology Department Yalgado Ouedraogo University Hospital Ouagadougou Burkina Faso

**Keywords:** bulbar ulcer, diagnosis, hematemesis, portal cavernoma

## Abstract

A portal cavernoma is a network formed by localized dilation of the hepatic portal system. This study aims to describe a case of portal cavernoma in a 69‐year‐old adult presenting with gastrointestinal hemorrhage. This is a 69‐year‐old patient with no particular medical history admitted for hematemesis associated with diffuse abdominal pain. Clinical examination revealed blood pressure of 100/60 mmHg and tenderness in the epigastric region. Upper gastrointestinal endoscopy revealed a large bulbar ulcer in flare‐up. *Helicobacter pylori* testing in stool samples was positive. The initial diagnosis was a bulbar ulcer, and the patient was placed on treatment to eradicate *H. pylori* . However, an abdominal ultrasound performed later suggested heterogeneous portal vein thrombosis, which was confirmed by abdominal CT scan. Given these findings, we made a diagnosis of portal cavernoma revealed by gastrointestinal hemorrhage. Treatment consisted of 40 mg of injectable omeprazole every 12 h, 40 mg of propranolol per day, and one sachet of Gaviscon every 8 h. Anticoagulation was initiated with enoxaparin and then continued with 4 mg of acenocoumarol per day. The patient responded well to treatment, with regression of abdominal pain and gastrointestinal hemorrhage, and was discharged home after 10 days following two stable INR readings. Although most commonly described in pediatrics, this clinical case sheds light on adult forms of the disease.

## Key Clinical Message

The identification of a peptic source during endoscopy does not rule out the coexistence of portal hypertension in adults presenting with upper gastrointestinal bleeding. When clinical signs suggest portal hypertension—splenomegaly, ascites, varices—abdominal imaging should be performed, as portal vein thrombosis with cavernous transformation may coexist and constitute the main risk factor for hemorrhage.

## 1 Introduction

A portal cavernoma is a network of veins, initially millimetric or microscopic in size, which increases in diameter and through which hepatoportal portal blood flows [1]. It is the result of chronic, often thrombotic occlusion of the extrahepatic portal system lasting more than 3 weeks [2]. It was first described in 1955 during the autopsy of a patient who died of mesenteric venous thrombosis [3]. However, since then, very few cases have been reported in the African literature, particularly in sub‐Saharan Africa [1]. In this study, we describe a case of portal cavernoma in a 69‐year‐old adult with gastrointestinal hemorrhage.

## 2 Observation

### 2.1 Medical History

This is a 69‐year‐old patient with no particular medical history. He was admitted for hematemesis associated with diffuse abdominal pain over the past 2 days, followed a day later by moderate diarrhea with blackish stools. There was no indication of consumption of food or toxic substances.

### 2.2 Physical Exam

Clinical examination revealed blood pressure of 100/60 mmHg, heart rate of 110 beats per minute, respiratory rate of 20 cycles per minute and weight loss with a body mass index of 18 kg/m^2^. There was tenderness in the epigastric region, moderate ascites, and Hackett stage II splenomegaly. Cardiac auscultation revealed regular tachycardia at 110 beats per minute, and the rest of the physical examination was normal.

### 2.3 Paraclinics Exams

Upper gastrointestinal endoscopy carried out shortly after he was admitted to hospital revealed a large bulbar ulcer (7–9 mm of diameter) in flare‐up. There were also grade IIa esophageal varices without red signs, and a mosaic appearance of moderate portal hypertension. The stool test for *Helicobacter pylori* was positive.

### 2.4 Initial Diagnosis

An initial diagnosis of a bulbar ulcer was made and the patient was placed on treatment to eradicate *H. pylori* .

### 2.5 Secondary Paraclinical Exams

The abdominal ultrasound performed 2 days later was consistent with heterogeneous portal vein thrombosis with abundant ascites and a normal‐sized, non‐dysmorphic liver. The abdominal CT scan performed with portal vein injection revealed a heterogeneous defect in the portal trunk and its branches, with upstream dilation, consistent with a thrombotic lesion of the portal trunk; the heterogeneous centre of the lesion did not take up contrast, suggesting a portal cavernoma. There was also thrombosis of the splenic vein (Figure 1). The spleen had a nodule suggestive of an angioma, and the pancreas was atrophic with a 9 mm dilation of the Wirsung duct. Biologically, transaminases were slightly elevated (AST = 44 IU/L and ALT = 48 IU/L). The blood ionogram, complete blood count and prothrombin level were normal. Retroviral serology for HIV and hepatitis B and C was negative. As part of the aetiological investigation, thrombophilia testing, particularly for protein C/S, antithrombin III, factor V Leiden and JAK2 mutation, could not be performed as these tests were not available in our setting.

**FIGURE 1:**
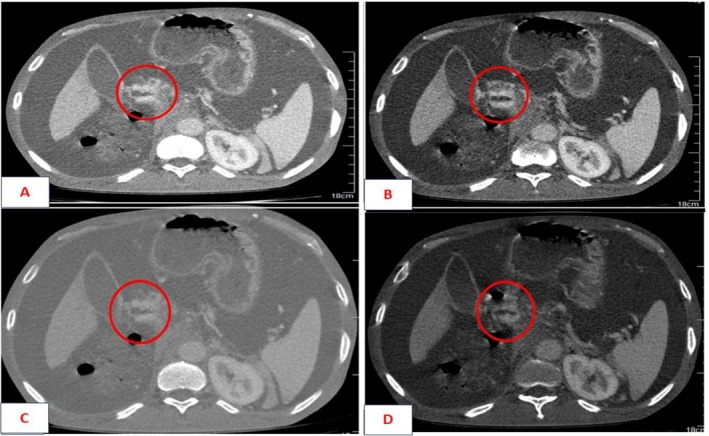
Abdominal computed tomography scan showing venous phase (early A and C, late B and D) revealing an excavated hyper density located at the portal vein (red circle) obstructing its lumen, causing upstream portal dilatation, suggesting a portal cavernoma (circled in red).

### 2.6 Diagnostic Return

Given this clinical picture, we diagnosed portal cavernoma revealed by gastrointestinal hemorrhage.

### 2.7 Treatment

Treatment consisted of 40 mg of injectable omeprazole every 12 h, 40 mg of propranolol per day, and one sachet of Gaviscon every 8 h. Following endoscopic band ligation and initial treatment with omeprazole, which successfully controlled the bleeding, anticoagulation was initiated with 0.8 mL of enoxaparin every 12 h, subsequently replaced with 4 mg of acenocoumarol. The target INR was 2–3.

### 2.8 Evolution

The patient's condition improved, with a reduction in abdominal pain, ascites, and gastrointestinal bleeding, and he was discharged home after 10 days following two stable International Normalized Ratio (INR) readings (2.3 and 2.5). No blood transfusion was required as the patient remained in a stable haemodynamic condition with a hemoglobin level of 11 g/dL. The patient was discharged from hospital with 40 mg of omeprazole (morning and evening) and 4 mg of acenocoumarol for a planned treatment duration of 6 months. He was seen again 3 months later and showed complete resolution of symptoms, with his INR remaining stable. However, at the six‐month follow‐up, the patient was lost to follow‐up and we were unable to obtain follow‐up imaging to assess the progression of the lesion.

## 3 Discussion

Portal cavernoma is rarely reported in our regions. In the United States, its incidence is estimated at 1% in the general population [4]. In Africa, its actual incidence is not well known but is thought to be less than 1% according to the literature. A Tunisian study in 2001 described 19 observations over a period of 25 years, while another study conducted in Morocco reported 11 cases between 2003 and 2012 [5, 6]. There is also a male predominance in most series and cases reported in the literature [1].

Sometimes discovered by chance, gastrointestinal hemorrhages are the main circumstances in which this condition is detected [1, 6, 7]. They are caused by the rupture of esophageal varices (OV) and constitute the severity of this pathology. Despite gastrointestinal hemorrhages, other clinical manifestations are neither constant nor specific. In line with the data in the literature, signs such as splenomegaly, abdominal pain, ascites, and transit disorders are frequently observed, as in our patient [8, 9]. In children, growth retardation is common and is thought to be linked to a decrease in hepatic portal flow and/or resistance to growth hormone [10].

Endoscopy revealed stage II esophageal varices without red signs. These varices are indicative of portal hypertension syndrome and are frequently encountered in 90% to 95% of cases [8]. Abdominal Doppler ultrasound is the first‐line examination for routine abdominal exploration and remains an excellent means of diagnosing and monitoring portal cavernomas. As in our patient, ultrasound data must be supplemented by a precise description of the size of the cavernoma and its impact on the mesenteric and splenomegaly systems [6, 11].

The exact etiology is unknown, but the authors believe that cavernoma is the result of chronic portal vein thrombosis. Indeed, the portal obstruction creates a network of collateral veins of varying caliber through which hepatoportal portal blood flows. In developing countries, omphalitis remains a common cause of portal thrombosis and therefore portal cavernoma, especially in children [12]. In adults, the onset of portal vein thrombosis is most often the result of a combination of local causes and requires systematic investigation for prothrombotic disease [8]. Portal hypertensive enteropathy and pancreatic atrophy are thought to be responsible for exocrine pancreatic insufficiency, which causes mucosal congestion and diarrhea; these symptoms are therefore part of a digestive malabsorption syndrome. Thrombophilia would also be difficult to rule out in this context, as the assessment could not be performed. Furthermore, an abdominal tumor could be a possibility, but this scenario is less likely as the abdominal CT scan has not revealed any suggestive findings.

In addition to anticoagulation, medical treatment with beta blockers allows for primary or secondary prevention of gastrointestinal hemorrhage due to rupture of esophageal varices [7]. Ligation and/or endoscopic sclerotherapy of varices will be indicated in case of failure, and a porto‐systemic shunt, with or without a porto‐biliary shunt, in case of biliary complications [9].

## 4 Conclusion

Portal cavernoma is a major cause of portal hypertension that is rarely described in adults. Although it is most often described in pediatrics', this clinical case allows us to shed light on adult forms in middle age. Its main manifestation is gastrointestinal hemorrhage, which is the most feared and serious aspect of this condition. In our context, management is limited to medical treatment, which is the entire problem in cases of catastrophic hemorrhage. This article reports an extremely rare case and serves as a reminder to clinicians of the need for greater vigilance in situations where the diagnosis may seem obvious.

## Author Contributions

**Martin Wendlassida Nacanabo:** conceptualization, data curation, investigation, methodology, writing – original draft. **André Arthur Taryètba Seghda:** supervision, validation. **Aimé Arsène Yaméogo:** supervision, validation. **Ella Hatoula Lengani:** supervision. **Adolphe Benjamin Zingué Ouattara:** supervision. **Aminata Guiarra:** supervision. **Yannick Laurent Tchenadoyo Bayala:** data curation. **Abdoul Nassir Porgo:** data curation. **Abdoul Aziz:** data curation. **Abdoul Djalilou Traoré:** data curation. **Anna Tall/Thiam:** supervision, validation. **Georges Christian Millogo:** validation. **Valentin Nobila Yaméogo:** supervision. **André Koudnoaga Samadoulougou:** validation, visualization.

## Funding

The authors have nothing to report.

## Consent

We have obtained the patient's written consent for the publication of this data. Confidentiality and anonymity have been respected.

## Conflicts of Interest

The authors declare no conflicts of interest.

## Data Availability

Data relating to this manuscript are available from the corresponding author.
